# Coordinating Diverse Functions of miRNA and lncRNA in Fleshy Fruit

**DOI:** 10.3390/plants12020411

**Published:** 2023-01-16

**Authors:** Reqing He, Yajun Tang, Dong Wang

**Affiliations:** 1 Key Laboratory of Molecular Biology and Gene Engineering in Jiangxi Province, College of Life Science, Nanchang University, Nanchang 330031, China; 2 Shandong Laboratory of Advanced Agricultural Sciences, Peking University Institute of Advanced Agricultural Sciences, Weifang 261325, China

**Keywords:** miRNA, long non-coding RNA, stress response, fruit, agricultural traits, CRISPR, molecular breeding

## Abstract

Non-coding RNAs play vital roles in the diverse biological processes of plants, and they are becoming key topics in horticulture research. In particular, miRNAs and long non-coding RNAs (lncRNAs) are receiving increased attention in fruit crops. Recent studies in horticulture research provide both genetic and molecular evidence that miRNAs and lncRNAs regulate biological function and stress responses during fruit development. Here, we summarize multiple regulatory modules of miRNAs and lncRNAs and their biological roles in fruit sets and stress responses, which would guide the development of molecular breeding techniques on horticultural crops.

## 1. Introduction

Fruits are differentiated from a mature ovary of the pistil after fertilization or floral organs and comprise a variety of pericarp and seed tissue types, which can be classified as dry or fleshy according to different pericarp textures [1]. Dry fruits or their seeds are usually derived from ovary tissues, such as *Arabidopsis thaliana*, which produces dry dehiscent fruits (silique) [2]. Fleshy fruits are derived mostly from hypanthium tissues that are hypothesized to consist of the fused bases of the sepals, petals and stamens [1], such as tomato (*Solanum lycopersicum*) and strawberry (*Fragaria × ananassa*). Fleshy fruits play important nutritious and health roles in the human diet, and several characteristics of the fleshy fruit, including color, flavor, aroma, texture and nutrition, have been studied for their dramatic changes during growth [3,4].

Likewise, although there is a remarkable convergence between fleshy fruit species, regardless of the ontogeny of the fruit, the processes involved in fleshy fruit development can be very different. For example, tomato fruit ripens in climacteric patterns, while strawberry fruit softening during ripening is accompanied by non-climacteric behavior [5]. Fruit development is under regulation at a complex molecular level and it explains the dynamics that alter the size, color, firmness, taste and flavor, which are characteristics intimately related to fruit quality. In addition, disease-resistant and stress-tolerant varieties also contribute an important part to fruit quality, fruit production and economic value. Fruit analysis provides insights into the potential for fruit crop improvement strategies and consequently applies to agricultural production [6,7].

More recently, a great number of functional genomics has shown that non-coding RNAs, especially miRNA and lncRNA, are involved in a diversity of developmental reproductive stages, from carpel formation and ovary development to the softening of the ripe/ripened fruit [8,9,10]. Moreover, numerous genetic studies have also shown that miRNA and lncRNA regulation results in fruit development alteration, including organ pattern, fruit shape and size, as well as their developmental progress, such as miR159 involved in fruit set [11], miR160 associated with fruit shape [12], miR164 affecting locule number [13,14] and miR156 regulating fruit softening [15]. This layer of transcriptional control has been associated with ovule, seed and fruit development and fruit ripening, as well as stress responses [16,17], which are crucial developmental processes in breeding programs because of their relevance for crop production. In addition, the final ripe fruit is the result of a process under multiple complicated levels of regulation that acts to coordinate the main steps of fruit development/patterning and fruit ripening, including mechanisms orchestrated by miRNA and lncRNA.

Although miRNA and lncRNA are particularly well studied and well known in fleshy fruit, the functions of miRNA involved in development processes are different in fruit species. In contrast, lncRNAs are usually less evolutionarily conserved, but the broad functions of lncRNAs are still possible under certain interactions. In this review, we discuss miRNA/lncRNA-controlled mechanisms described in the current literature that act to coordinate the main steps of fruit development/patterning, fruit ripening and fruit stress responses. Moreover, we discuss the main aspects of agronomic traits in fleshy fruits, such as yield (fruit size, fruit set), quality (parthenocarpic fruit) and stress tolerance, and explore the outlook for uses of miRNA/lncRNA-associated traits in fruit biotechnology and fruit breeding. In such a way, we present the latest advances in miRNA and lncRNA validation and the functional analysis as strong evidence for the regulatory roles in fruit biology (Table 1).

## 2. Functions of miRNAs and lncRNAs in Fruit Development

**Fruit size and/or fruit number** are crucial for improving yield and have a positive impact on consumer preference. It has been reported that the genes controlling tissue identity are involved in modulating fruit morphology, such as MADS-box genes, which are known to regulate floral organ identity, thereby regulating fruit development in *Arabidopsis* [18]. The regulatory module of miR172-*AP2* has been highlighted in regulating fruit development in diverse plants. In *Arabidopsis*, miR172 promotes the silique fruit expansion process by the negative regulation of the activity of the *APETALA2-like* (*AP2*) gene [19], which would otherwise hinder the action of *AGAMOUS* (*AG*) and *FRUITFUL* (*FUL*) [20], two MADS-box transcription factors that are essential for ovary and silique growth [21]. miR172 has an adverse influence on fruit size in apples (*Malus × domestica*) through the negative regulation of *AP2* that is required for hypanthium development into a pome fruit [22], resulting in small fruit size and an abnormal floral organ [23]. Another study in tomato, an ovary-derived fleshy fruit, revealed that the *SlMIR172c* and *SlMIR172d* loss-of-function mutant lines (*slmir172c-d^CR^*) resulted in abnormal flower organ and number identity [24]. These findings suggested a different role of miRNAs in dry and fleshy fruit. miRNAs regulate endogenous genes to impact development responses and even drive crop domestication; these results are consistent considering that silique is a true fruit deriving from ovary tissues, while the pome is a false fruit developing mainly from extra-carpellary tissues, such as sepals.

*MIR172* encodes highly similar miR172s, but exhibits differences in their distribution among fruit growth. These different biological functions and miRNA patterns in *Arabidopsis*, apple and tomato indicate the parallel evolution of the miRNA machinery in different fruit types. There are seven genes (*SlMIR172a–g*) that code for four unique species of miR172 (sly-miR172) in tomato [25], fifteen genes (*mdm-MIR172a–o*) in apple [26] and five genes (ath-miR172a–e) in *Arabidopsis* [27]. A recent study shows that the whole genome duplication (WGD) event of *Populus trichocarpa* stimulates the emergence of new miRNAs [28]. The number variety of miRNAs in different species may have resulted from the whole genome duplication event, thus contributing to the functional specialization of miRNAs and the functional importance of *MIR* genes. Many miRNAs are species-specific rather than conserved, which supports fruit type-specific divergence in miRNA evolution. Divergence in miRNAs or targets may have played important roles in horticultural crop domestication; for example, a loss-of-function mutation in *MIR172p* improved fruit size during apple domestication [22]. 

In addition, it has been observed that *sly-MIR156a-c* expressed in placenta, ovules and pre- and post-anthesis flowers in tomato [29], when overexpressing miR156a–c, resulted in the enhancement of vegetative development, a delay in flowering time, and a smaller number of fruits that presented ectopic leaf-like structures [15]. Moreover, the overexpression of tomato miR156 altered the expression of miR164, which is related to organ identity as well as carotenoid biosynthesis [15], suggesting that the miRNA–miRNA crosstalk and other molecular networks are also involved in fruit development. It has also been reported that miR156–miR172 pairs perform a negative correlation in flowering induction in *A. thaliana*, *Nicotiana tabacum*, *D. glomerate* and *Oryza sativa* [30,31,32,33], suggesting that miRNA–miRNA crosstalk plays an important role in the development of plant sexual organs.

**Figure 1:**
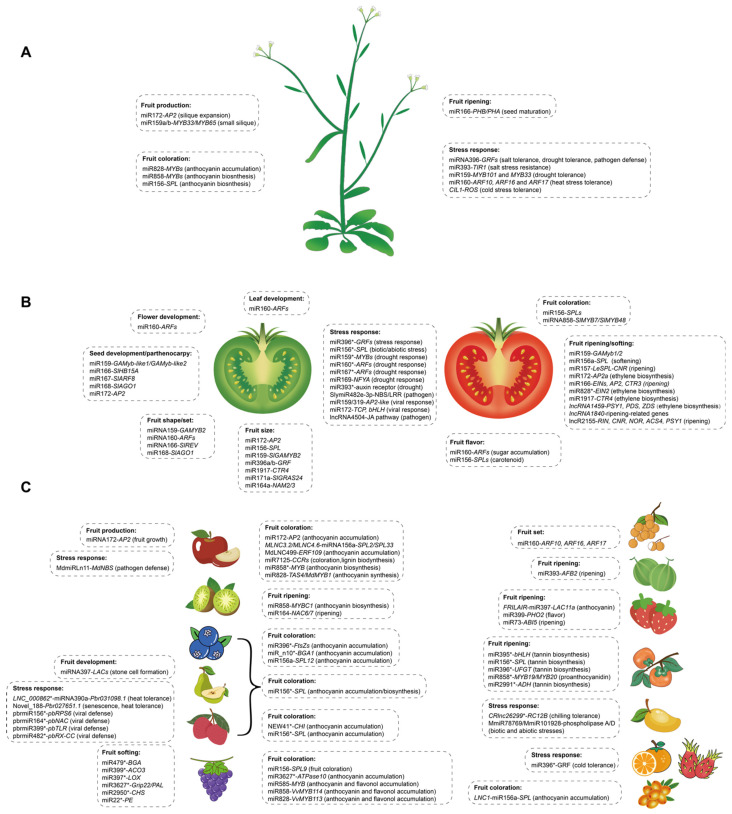
Diverse function of miRNA and lncRNA members in fruit crops: (**A**) *Arabidopsis*, (**B**) tomato and (**C**) fruit crops (fruit species on left: apple, kiwifruit, blueberry, pear, litchi, grape; fruit species on right: longan, melon, strawberry, persimmon, mango, orange, pitaya, and sea buckthorn). The asterisk (*) represents bioinformatics analysis data.

Furthermore, other miRNA regulation modules have also been identified in regulating fruit size and number (Table 1, Figure 1A,B). The sly-miR171a gene regulates hormone crosstalk between auxin and gibberellin in fruit size/weight by targeting two members of the GRAS family (*SlGRAS24* and *SlGRAS40*) known as hormone regulators [34]. In this way, *SlGRAS24* silencing results in GA3 and IAA accumulation, which leads to cell division and cell growth, and then floral initiation and seed number alteration [35,36]. Furthermore, sly-miR396a-3p/5p and sly-miR396b are mainly expressed in fruit, highlighting their potential role in fruit development. Knocking down miR396 by short tandem target mimic (STTM) showed an increase in fruit weight (66%), sepal size (153%), cell number (99%) and size (65%) [37], suggesting that the attenuation of miR396 results in the enhancement of some key performance indicators for fruit production. It has also been observed that knocking out miR164a by CRISPR/Cas9 to release the expression of *NAM2/3* leads to decreased tomato fruit size [38]. The knocking down of miR1917 targeting an ethylene response gene *CTR4* in tomato leads to bigger fruit [39]. It is reported that sly-miR159 is essential for fruit growth in *Arabidopsis*, and the *mir159ab* double mutant leads to small siliques [40], while its silencing results in larger fruits in tomato [41], suggesting fruits developed from the ovary may have evolved a different role of miRNAs in dry and fleshy fruit.

**Fruit set or fruit shape** is a crucial indicator of fruit development affecting the economic values of fruit and consumers’ preferences. During the initial fruit growth phase, rapid growth in fruit length and width, mainly due to rapid cell division and expansion driven by phytohormones such as auxin and gibberellins (GAs), is observed in the tissues surrounding fertilized ovules [42]. It has long been known that auxin is involved in plant growth as well as development and fruit set [43,44,45]. Early studies revealed that the knockdown of sly-miR160a by STTM technology resulted in the alteration of floral organ abscission and auxin-mediated ovary patterning as well as fruit shape through the post-transcriptional regulation of the auxin response factors *ARF10A*, *ARF10B* and *ARF17* [12]. The overexpression of *ARF10A* resulted in greener fruits before the BR stage, enhanced photosynthesis rate and sugar accumulation [12,46]. These results suggest the important roles of sly-miR160 auxin-mediated fruit shape and sugar accumulation in tomato [47].

Moreover, many transcript factors (TFs) are miRNA targets that regulate key genes involved in the floral induction and flower formation processes such as transition phases from juvenile to adult, the initiation of floral competence and flower development [48]. A large number of species-specific miRNAs have also been identified in tomato fruit development [49]. For example, silencing miR159 induced more locules inside the tomato fruit [11]. The miR159–*SlGAMYB1/2* module is involved in tomato ovary development and fruit set by modulating auxin and gibberellin responses during ovule and ovary development [11]. Additionally, the overexpression of an miR166-resistant mutant of *REV* (35S::REV*^Ris^*) results in ectopic fruits on receptacles and fused fruits [50], indicating that the functioning of *REV* mainly depends on the regulation of miR166 at the posttranscriptional level. In addition, is reported that the overexpression of PbrmiR397a reduced the lignin content and stone cell number in pear fruit (*Pyrus bretschneideri*) by inhibiting laccase (*LAC*) genes that encode key lignin biosynthesis enzymes [51]. The genome analysis of a single nucleotide polymorphism (SNP) in the promoter of *PbrmiR397a* between sixty pear varieties was found to be associated with low levels of fruit lignin [51]. Moreover, miR160 regulates longan somatic embryo development by targeting *ARF10*, *ARF16* and *ARF 7* [52]. Altogether, these results unveil the function of the miRNA-targeted key genes in regulating an agronomically important trait.

In addition, transcriptome-wide analyses have revealed the important regulatory roles of lncRNAs in a set of fruit developmental processes, such as lncRNAs function in regulating flower and fruit development in strawberry [53], peach (*Prunus persica*) [54] and apple [55]. Although many lncRNAs have been identified in diverse fruit species during fruit developmental stages, their biological functions will be fully elucidated in the future (Table 1, Figure 1A–C).

**Parthenocarpic or seedless fruits** are an important agricultural trait and a desirable trait for consumers. Methods of achieving seedless/parthenocarpic fruits have received increasing attention from researchers and breeders. Fruit and seed development held a distinct place in plant propagation and harvesting for defining crop yields; therefore, the roles played by the miRNAs in the development of fruits and seeds are of great interest. In tomato, the interaction of AGO1s–miR168 not only determines fruit initiation and growth, but also exhibits parthenocarpy in miR168-resistant *4m-SlAGO1A* [56]. The overexpression of miR159 induced parthenocarpy as well as the downregulation of miR167, releasing the expression of *SlARF8* [11]. It was also observed that the *SlHB15A* transcript is recessive-dosage-sensitivity-controlled by miR166, and *Slhb15a* knockdown alleles by miR166 lead to a parthenocarpic fruit set [57]. The *MIR172* gene loss-of-function mutant line (*slmir172c-d^CR^*) produces an abnormal ovary expanded to form small parthenocarpic fruit-like organs [24]. 

It is reported that the molecular mechanisms of parthenocarpic fruit formation are mainly related to plant hormones, for example, cytokinin-treated pear (*Pyrus* spp.) and kiwifruit (*Actinidia chinensis*) [58,59], gibberellic acid-treated and auxin-induced tomato [60,61], brassinosteroids-induced apple [62], cytokinin-regulated and auxin-regulated grape (*Vitis vinifera*) [63] and gibberellic acid-maintained citrus (*Citrus clementina*) [64]. It is well established that parthenocarpy/seedless work in various fruits contributes to improving the taste quality of the fruit.

## 3. Functions of miRNAs and lncRNAs in Fruit Ripening

**Fruit color** variation is the most important agricultural trait of fruit ripening and chiefly affects the postharvest texture and consumers’ preferences. miRNAs have been extensively investigated in fruit development, and they also play an important role in fruit ripening. Interestingly, some miRNA regulations work in the same biological processes during fruit ripening. It has been found that the miR156-SPLs [65,66,67] and miR828/858-MYBs [68,69,70,71,72] modules are the conserved pathways to regulate fruit coloration in various fruit crops. For example, the miR156a-SPL12 module manipulates the accumulation of chlorophylls and anthocyanins during fruit ripening in blueberry, in which *VcSPL12* interacts with *VcMYBPA1* [66]. In pear, miR156-targeted SPLs interfere with the MYB-bHLH-WD40 complex in anthocyanin biosynthesis [73]. The transient overexpression of VvmiR156b/c/d in tomato promotes fruit coloring by repressing *VvSPL9* transcription [74], suggesting that VvmiR156b/c/d-mediated *VvSPL9* is involved in the formation of grape color. Similarly, in litchi (*Litchi chinensis* Sonn.), miR156a-targeted *LcSPL1*, interacting with *LcMYB1*, functions as a major cue in anthocyanin biosynthesis [67]. Moreover, the overexpression of miR156 promotes the accumulation of anthocyanins by targeting *SPL9* in *Arabidopsis*, which negatively inhibits anthocyanin biosynthetic genes through the destabilization of an MYB-bHLH-WD40 transcriptional activation complex [65]. Another study reveals that long non-coding RNA MLNC3.2 and MLNC4.6 function as eTMs for miR156a and suppress the miR156a-mediated cleavage of *SPL2-like* and *SPL33* during light-induced anthocyanin accumulation in apple fruit [75]. Similarly, the lncRNA *LNC1*-induces the downregulation of *SPL9* through endogenous target mimics of miR156a, which leads to increased anthocyanin content in sea buckthorn (*Hippophae rhamnoides* Linn.) [76]. Moreover, ncRNAs associated with the anthocyanin biosynthesis pathway have also been reported in various regulatory modules, such as NEW41-*CHI* in litchi [67], miR396-*FtsZs* and miR_n10-*BAG1* associated with blueberry [77] and miR172-*AP2,* miR7125-*MYB16/MYB1* and MdLNC499-*ERF109* involved in apple [78,79,80], all of which have been identified in anthocyanin accumulation (Table 1).

In addition, miR828 triggers the biogenesis of phasiRNAs that, in trans or in cis, regulate multiple *MYBs* that are involved in anthocyanin accumulation [8,68,69]. These MYBs belong to the R2R3 class, which is integrated with multiple biological processes, particularly in plant anthocyanin biosynthesis [81]. In *Arabidopsis*, the overexpression of miR828 reduces anthocyanin accumulation by repressing genes encoding *MYB* transcription factors [68]. In tomato, miR858 plays a negative role in anthocyanin biosynthesis, and the blockage of miR858 leads to increased anthocyanin accumulation by modulating the expression of *SlMYB7* and *SlMYB48* [70]. Another report demonstrates that miR858a represses the translation of *MYBL2* in *Arabidopsis* seedlings as a positive regulator of anthocyanin biosynthesis [72]. In grape, miR828/miR858 targets *VvMYB114*, which is reported as being essential for anthocyanin and flavonol accumulation [69]. The transient overexpression of miR858 reduces anthocyanin accumulation in kiwifruit (*Actinidia arguta*) by repressing the target gene *MYBC1* [71]. Among them, miR828 and miR858 could directly or indirectly control anthocyanin biosynthesis in apple [26]; for example, a recent study found that the overexpression of mdm-miR828 inhibited anthocyanin synthesis through the cleavage of *MdTAS4* in the late fruit coloration stage, and *MdMYB1* was induced in a feedback regulatory mechanism through binding to the promoter of *mdm-MIR828b* to promote its expression [82].

**Fruit ripening** is a complex biological process and is associated with many aspects of fruit flavor and textural alterations. In persimmon (Diospyros kaki Thunb.), miR395p-3p and miR858b regulate *bHLH* and *MYB*, respectively, which synergistically regulate the structural genes responsible for tannin biosynthesis [83]. In addition, many miRNAs’ target genes have been identified through high-throughput sequencing associated with regulating persimmon fruit ripening, such as miR156-*SPL*, miR396-*UFGT*, miR858-*MYB19/20* and miR2991-*ADH* [83]. Another study on strawberry shows that the overexpression of miR399 can improve fruit quality by targeting *PHO2* [84]. A novel miRNA, Fan-miR73, negatively regulates its target gene, *ABI5*, to control strawberry fruit ripening [85]. Knocking down pre-slymiR157 or mature slymiR157 delays tomato fruit ripening by targeting *LeSPL-CNR*, in turn regulating the expression of *LeMADS-RIN*, *LeHB1*, *SlAP2a* and *SlTAGL1* [86,87]. Additionally, miR164-*NAC6/7* and miR393-*AFB2* are associated with fruit ripening in kiwifruit and melon (*Cucumis melo*) [88,89] (Table 1). These sophisticated regulatory networks might provide the accurate regulation of fruit ripening in different plants.

LncRNAs also play important roles in the fleshy fruit-ripening process. The genome-wide discovery and characterization of novel species-specific lncRNAs in fruits were conducted in various fleshy fruit species, including tomato [10,90], strawberry [53], apple [91], grape [92], kiwifruit [93], peach, mume (*Prunus mume*) [94], sea buckthorn [95] and melon [96]. These results present the global function of lncRNAs in different fruit species, which provides new insights into the regulation of fruit quality.

In strawberry, color change in wild varieties of *Fragaria pentaphylla* (*F. pentaphylla*) may be largely regulated by lncRNAs [97]. In tomatoes, silencing two lncRNAs, *lncRNA1459* and *lncRNA1840*, delayed the fruit-ripening processes, which indicated the positive regulatory roles of the two members in the fruit-ripening process [10]. Furthermore, knocking down *lncRNA1459* by CRISPR/Cas9 genome editing technology affected lycopene, carotenoid and ethylene biosynthesis [17]. Moreover, in tomato, 187 lncRNAs were found to be direct targets of the MADS-box transcription factor (TF) *RIPENING INHIBITOR* (*RIN*), which is a critical TF of fruit ripening [98,99]. In the fleshy fruit species, lncRNAs were also reported to be the key regulators with miRNAs under sophisticated control to perform their proper function. Some research has shown that long non-coding RNA (lncRNA) could regulate miRNAs as endogenous target mimics (eTMs) and participate in anthocyanin accumulation, such as MLNC3.2 and MLNC4.6 in apple [75] and *LNC1* in sea buckthorn [76]. In strawberry, the lncRNA *FRILAIR* serves as a miRNA sponge by functioning as a noncanonical target mimic of strawberry miR397, which can guide the mRNA cleavage of the fruit-ripening accelerating gene *LAC11a*, thereby regulating the fruit-ripening process [100]. Knocking out lncRNA2155 by CRISPR/Cas9 technology delayed tomato fruit ripening with downregulated ripening-related genes, including *RIN*, *CNR*, *NOR*, *ACS4* and *PSY1* [98].

**Fruit softening and fruit texture** are also crucial for optimizing fruit quality. In addition, several ncRNAs are involved in fruit softening. Knocking down pre-miR156a–c or their mature SlymiR156a sequences through the VIGS system accelerates tomato fruit softening after the red ripe stage [87]. Additionally, miR479-*BGA*, miR2950-*CHS*, miR22-*PE*, miR3627-*PAL* and miR399-*ACO3* are associated with fruit softening in grapes [101] (Table 1). Furthermore, the overexpression of miR399a can promote the accumulation of fructose and glucose in wild strawberry fruit [84]. In apple, the overexpression of miR7125 reduces lignin biosynthesis by targeting *MdCCR* during light induction [79]. Taken together, it will be important to extensively explore the underlying mechanisms in fruit ripening.

**Plant hormones** in fruit ripening are necessary, and the molecular mechanism and the signaling cascades of plant hormones during fruit ripening have been extensively studied in horticultural plants [102]. Non-coding RNAs are also involved in phytohormone regulation networks, such as ethylene (ETH), which is the major phytohormone in climacteric fruit ripening [103,104]. Tomato miR172 targets *SlAP2* cleavage to accelerate fruit ripening and enhance ethylene biosynthesis [105]. Furthermore, slymiR1917 was reported as a negative regulator of two ET-related *CTR4* splicing variants, but it is also regulated to *ACS2* and *ACS4*, which are key genes for the establishment of the type of ET synthesis pathway [106]. In particular, *Ethylene Insensitive 2* (*EIN2*) is targeted by miR828 [107], therefore for the onset of ethylene-dependent ripening events, a strong reduction of the expression of both miR394 and miR828 is required in tomato [108]. Moreover, some other miRNAs were found as regulators of some ET-related genes, such as the overexpression of the miR166-resistant version of *SlREV* downregulating *EIN3*, *ERFs*, *AP2* and *CTR3* in tomato [52]. The interplay may provide a mechanism to enable flexible fruit ripening. Several different types of non-coding RNAs are involved in regulating the expression of ripening genes, but further clarification of their diverse mechanisms of action is required. Further investigation might help to understand whether this behavior is relevant for development and if there are some other offset mechanisms in terms of time to ripen.

## 4. Functions of miRNAs and lncRNAs in Fruit Responses to Biotic and Abiotic Stress

Stress tolerance is an important breeding objective and selection criteria in breeding that is critical for fruit quality, such as disease-resistant varieties, cold-resistant varieties and drought-resistant varieties. Besides the role of miRNAs and lncRNAs in growth, development and ripening, they also act as important signaling components in stress responses. They are key modulators of the transcriptional and post-transcriptional expression of genes during defense responses, and they are shown to be required for adaptation to the changes in ambient environments. Stress-induced changes occur in multiple species and correlate with a conserved mechanism involving non-coding RNA regulations. 

**Salinity stress** usually causes physiological disorders in fruit crops. During salinity conditions, numerous gene transcripts are variably regulated by miRNAs. The auxin signaling plays an important role in the biotic stress response, and the miR393-mediated regulation of the auxin receptor *TIR1* is involved in the response to salt stress resistance and ABA-signaling pathways [109,110]. Furthermore, the miR396-GRF module was shown in pitaya (*Hylocereus polyrhizus*) and *Arabidopsis* [111,112]. Interestingly, a wide range of miRNAs was induced in date palms (*Phoenix dactylifera* L.) and mandarin (*Citrus reticulata Blanco*) under salt stress conditions [113,114], which provides insight into plants’ adaptation to salinity.

**High or low temperature stress** at the fruit development stage is an important factor that determines fruit quality and fruit storage time, and hot or cold temperatures influence plant growth and yields. Several miRNAs induced by high-stress conditions have been identified through the bioinformatic prediction or RLM-5′ RACE-based validation in tomato, suggesting that a miRNA-mediated regulatory network is involved in high temperature [115]. In *Arabidopsis*, miR160 repressed *ARF10*, *ARF16* and *ARF17* to release the expression level of heat shock protein genes to allow the plants to survive heat stress [116], while miR160-*ARF18* mediated salt tolerance in peanut [117]. In pear, a novel miRNA, Novel_188, is validated to target *Pbr027651.1* to mediate fruit senescence under high- or low-temperature conditions [118]. Ptr-miR396b was determined to target *1-aminocyclopropane-1carboxylic acid oxidase* (*ACO*) in response to cold stress in orange (*Poncirus trifoliata*) [119]. In mango, bioinformatic analysis reveals that MmiR78769 and MmiR101928 target phospholipase A and phospholipase D, respectively, both of which are associated with plant temperature stress-responsive process [120]. Moreover, degradome-wide analyses have revealed that miR393-*TIR1*/*AFB* displays a cold stress-specific response and miR156-*SPL*-mediated heat stress response in banana [121]. 

In particular, the lncRNAs’ temperature stress responses were found to be very specific. High temperature-induced *LNC_000862* is likely to delay pear fruit senescence by competing with miR390a to derepress the expression of *Pbr031098.1* [122]. LncRNAs involved in the response of chilling injury in tomato fruit have been systematically identified, providing a new perspective on lncRNA roles in chilling tolerance in fruits [123]. In mango, the cold-responsive lncRNA *CRlnc26299* can interact with *RC12B*, which is the low-temperature and salt-responsive protein [120]. A novel lncRNA, *COLD INDUCED lncRNA 1* (*CIL1*), is a positive regulator in plant response to cold stress by regulating the expression of endogenous reactive oxygen species (*ROS*) in *Arabidopsis* [124].

**Drought stress** adversely affects fruit crops’ productivity and quality. Drought stress response modulation via the miRNA pathway has also been found in several plant species. In tomato, miR159, miR169, miR160, miR167 and miR393 are associated with dehydration stress tolerance, by controlling hormonal signal transduction, stomatal closure and auxin-responsive genes [125,126]. The overexpression of miR396 showed lower densities of stomata and induced drought tolerance in *Arabidopsis* by suppressing the expression of *GRF* [127], which was consistent with the finding that the miR396-*GRF* module is involved in stress tolerance in tomato and pitaya [112,125]. Moreover, ABA-induced miR159 inhibits the transcripts of *MYB101* and *MYB33* during seedling stress responses in *Arabidopsis* [128]. A novel lncRNA, named *DROUGHT INDUCED lncRNA* (*DRIR*), has a positive role in the response of *Arabidopsis* to drought and salt stress [129].

**Pathogen defense** is associated with fruit quality and postharvest quality. Plants are constantly exposed to a range of microbial pathogens with different lifestyles and modes of attack, including fungal, bacterial and viral pathogens, whereas RNA-based mechanisms largely regulate plant–virus interactions. Many key miRNA regulators of the stress response in fruits during pathogen infection were identified, such as miRNAs engineer *Botrytis cinerea* (*B. cinerea*) in kiwifruit [130] and specific miRNAs’ response to stress in Amur grape (*vitis amurensis Rupr.*) [131]. In particular, Md-miRLn11 targeted an apple nucleotide-binding site (NBS)–leucine-rich repeat (LRR) class protein coding gene (*Md-NBS*) to trigger host immune responses during pathogen infection [132]. SlymiR482e-3p knocking-out lines showed enhanced resistance to tomato wilt disease and regulated ethylene signaling by suppressing the expression of ethylene response factors *(SlERFs*) [133]. The can-miRn37a further confirmed anthracnose resistance in chili (*Capsicum annuum* L.) by repressing *ERFs* and preventing fungal colonization [134]. In tomato, sly-MIR156d/e were found induced under biotic and abiotic stress [29,135]. In addition, miR159/319 and miR172 accumulation positively correlated with immune responses during *Tomato leaf curl virus* (ToLCV) infection, indicating that miR159/319 and miR172 might be associated with the response to viral infection in tomato [136]. In pear, pbr-miR156, pbr-miR164, pbr-miR399 and pbr-miR482 are induced during *Apple stem pitting virus* (ASPV) infection and then trigger its target genes to participate in viral defense pathways [137]. Overexpressed miR396 not only plays roles in drought response in *A. thaliana* [127] and cold tolerance in orange [119], but also has resulted in plant tolerance under the attack of necrotrophic fungal pathogens [138]. Previous reports have shown that lncRNA not only plays essential roles in diverse biological processes, but also in various stress responses. LncRNA4504 positively regulated methyl jasmonate (MeJA)-induced tomato fruit resistance to *B. cinerea* by promoting the accumulation of total phenols and flavonoids and upregulating the expression of JA signal pathway genes [139].

The most effective postharvest technology to maintain fruit quality is to delay the fruit senescence process, such as cold storage after the fruit is harvested. Thus, in incorporating the dynamic environments, important alterations in non-coding RNA transcriptomes are observed in many plant species, which has led to the general view that plants utilize ncRNAs as part of their arsenal to cope with the wide array of microbial pathogens they encounter (Table 1). Further investigation might help to find clues to a better understanding of the consequences of ncRNA attenuation under biotic and abiotic stress and its putative success under field conditions.

**Table 1:** miRNAs and lncRNAs with functional verification in fruit crops.

Fruit Biology	Classification	Species	Non-Coding RNA	Targets/Downstream	Functionally in Fruit Quality	Research Methods	References
Fruit development	Fruit size and number	arabidopsis	miR172C	*APETALA2-like*	silique fruit expansion	stable (MIR172C::GUS, MIR172C^AuxRE^::GUS)	[21]
			miR159a/b	*MYB33/MYB65*	altered growth habit, curled leaves, small siliques, and small seeds	T-DNA mutants (*mir159ab* double mutant)	[40]
		apple	miR172p	*AP2*	reduced fruit size, altered floral organ development	stable (*MIR172p* OE in tomato)	[22,23]
		tomato	miR156	*SPL*	fruit growth, ovary and fruit development	stable (*AtMIR156b* OE)	[15]
			miR159	*SlGAMYB2 (GA biosynthesis gene)*	larger fruits	STTM-miR159	[41]
			miR172d	*AP2*	floral organ identity and number	CRISPR/Cas9 (*slmir172c-d^CR^*)	[24]
			miR396a/b	*GRF*	a larger plant, with bigger flowers, leaves, and fruits	STTM-miR396	[35,36,37]
			miR1917	*CTR4* (altered ethylene response)	fruit size, bigger fruit	STTM-miR1917	[39]
			miR171a	*SlGRAS24 and SlGRAS40* (altered gibberellin and auxin)	cell number and size, smaller tomato fruit	*GRAS24* OE	[34]
			miR164a	*NAM2/3*	decreased fruit size	CRISPR/Cas9 (*slmir164a, slmir164b, slmir164d, slmir164^CR^*)	[38]
Fruit development	fruit set	tomato	miR159	*SlGAMYB2* (GA biosynthesis gene)	fruit morphology, precocious fruit initiationflattened, fruit with more locules inside	SlMIR159 OE	[11]
			miR160	*ARF10, ARF16* and *ARF17*	sugar accumulation, leaf and flower development, somatic embryo development, pear-shaped fruit	STTM-miR160	[12,46,47]
			miR166	*SlREV*	fruit formation	Overexpression of a microRNA166-resistant version of *SlREV* (35S::REV^Ris^)	[50]
			miR168	*SlAGO1s*	fruit initiation and growth	miR168 loss-of-function (four-point-mutated miR168-resistant *4m-SlAGO1A* and *4m-SlAGO1B)*	[56]
		pear	PbrmiR397a	*LACs*	stone cell formation, reduced lignin content and stone cell number	transient (*PbrmiR397a* OE, pear), stable (*PbrmiR397a* OE, tobacco)	[51]
		longan	miR160	*ARF10, -16,* and *-17*	somatic embryo development	target mimics down-regulate miR160	[52]
	seed development/parthenocarpy	tomato	miR159	*GAMyb-like1* and *GAMyb-like2*	parthenocarpy	*SlMIR159* OE	[11]
			miR166	*SlHB15A*	parthenocarpic fruit set	used TILLING to screen for *SlHB15A* miR166-resistant alleles	[57]
			miR167	*SlARF8*	parthenocarpy	downregulation of miR167	[11]
			miR168	*SlAGO1s*	parthenocarpy	miR168-resistant *4m-SlAGO1A*	[56]
			miR172	*AP2*	small parthenocarpic fruit-like organ	CRISPR/Cas9 (*slmir172c-d^CR^*)	[24]
Fruit ripening	fruit color	litchi	miR156a *	*LcSPL1/2*	anthocyanin biosynthesis	High-Throughput Sequencing and Degradome Analysis	[67]
			NEW41 *	*CHI*	anthocyanin accumulation		
		pear	miR156 *	*SPL*	Red Peel Coloration, anthocyanin biosynthesis	Degradome Library	[73]
		blueberry	miR156a	*VcSPL12*	anthocyanin accumulation	*VcMIR156a* OE in tomato	[66]
			miR396 *	*FtsZs*	coloration	Small RNA and Degradome Sequencing	[77]
			miR_n10 *	*BAG1*	coloration		
		apple	miR172	*AP2-MYB10*	flavonoidse, reduction in red coloration	miR172 OE	[80]
			MLNC3.2 and MLNC4.6 (lncRNA)	miR156a-*SPL2-like* and *SPL33*	anthocyanin biosynthesis	transient (35S::MLNC3.2, 35S::MLNC4.6, OE-miR156a)	[75]
			miR7125 (light-induced)	*MYB16/MYB1-CCRs*	promoted anthocyanin synthesis, reduced lignin biosynthesis	transient (miR7125 OE)	[79]
			MdLNC499 (lncRNA)	*MdERF109*	fruit coloration	transient (TRV-MdLNC499, TRV-*MdERF109*, apple fruit), stable (MdLNC499 OE, MdLNC499 RNAi, *MdERF109* OE, *MdERF109* RNAi, apple calli)	[78]
			mdm-miR828	*TAS4-MdMYB1*	inhibited anthocyanin synthesis	transient (*mdm-miR828* OE, apple, stable (*mdm-miR828* OE, *Arabidopsis*)	[82]
			miR858 *	*MYB*	anthocyanin biosynthesis	small RNA-seq	[26]
		sea buckthorn	*LNC1* (lncRNA)-miR156a	*SPL9*	anthocyanin accumulation	transient (TRV-*LNC1*)	[76]
Fruit ripening	fruit color	grape	miR858	*VvMYB114*	anthocyanin and flavonol accumulation	Degradom, transient/stable (*VvMYB114* OE, tobacco)	[69]
			miR156	*SPL9*	promoted fruit coloration	*miR156b/c/d* OE in tomato	[74]
			miR3627 *	calcium-transporting ATPase10	anthocyanin accumulation	sequencing small RNAs, bioinformatics analysis	[101]
			miR828	*VvMYB113/VvMYB114*	anthocyanin and flavonol accumulation	*vvi-miR828* OE, *Arabidopsis*	[69]
		arabidopsis	miR828	*MYB75, MYB90, and MYB113*	anthocyanin accumulation	*AtmiR828* OE	[68]
			miR858a	*MYB2*	anthocyanin accumulation, anthocyanin biosynthesis	STTM-miR858	[72]
			miR156	*SPL9* and *SPL15*	anthocyanin biosynthesis	*MIR156b* OE	[65]
		tomato	miR858	*SlMYB7* and *SlMYB48*	anthocyanin accumulation	STTM-miR858	[70]
		kiwifruit	miR858	*AaMYBC1*	anthocyanin biosynthesis	transient (*miR858* OE)	[71]
	fruit ripening, fruit softening and fruit quality	persimmon	miR395 *	*bHLH*	tannin biosynthesis	high-throughput sequencing	[83]
			miR156 *	*SPL*	tannin biosynthesis		
			miR396 *	*Flavonoid 3-O-glucosyltransferase* (*UFGT*)	tannin biosynthesis		
			miR858 *	*MYB19/20*	reduced the content of proanthocyanidin (PA)		
			miR2991 *	*ADH*	tannin biosynthesis		
Fruit ripening	fruit ripening, fruit softening and fruit quality	strawberry	*FRILAIR* (lncRNA)-miR397	*LAC11a*	delayed fruit ripening	transient (*miR397* OE, Cas13b-miR397, ocotoploid strawberry)	[100]
			fan-miR73	*ABI5*	fruit ripening	5′ -RACE analysis	[85]
			miR399	*PHO2*	flavor, sugar content	miR399a OE (woodland strawberry)	[84]
		tomato	miR157	*SPL-CNR*	delayed fruit ripening	*miR157* OE	[86,87]
			miR156	*SPL*	accelerates tomato fruit softening	VIGS-miR156a	[87]
			miR172	*AP2a*	accelerates fruit ripening with enhanced ethylene biosynthesis	*miR172* OE	[105]
			miR166	*SlREV*	fruit ripening	*35S::REV^Ris^* (*EIN3, ERFs, AP2,* and *CTR3* downregulated)	[50]
			miR828 *	*EIN2*	ethylene-dependent ripening	high throughput sequencing	[108]
			miR1917	*CTR4*	enhances ethylene response and accelerates fruit ripening	*miR1917* OE	[107]
			lncRNA2155 (lncRNA)	*RIN, CNR, NOR, ACS4, PSY1*	delayed fruit ripening	CRISPR/Cas9 (lncRNA2155 KO)	[98]
			*lncRNA1459* (lncRNA)	*PSY1, PDS, ZDS*	ripening, ethylene biosynthesis	CRISPR/Cas9 (*lncRNA1459* KO)	[10,17]
			*lncRNA1840* (lncRNA)	ripening-related genes	ripening, ethylene biosynthesis	TRV-*lncRNA1840*	[10]
		kiwifruit	miR164	*NAC6/7*	fruit ripening	*miR164* OE (kiwifruit callus)	[88]
		apple	miR7125	*MYB16/MYB1-CCRs*	reduced lignin biosynthesis	transient (miR7125 OE, apple fruit)	[79]
		melon	cme-miR393	*CmAFB2*	delayed fruit ripening	cme-miR393-OE	[89]
Fruit ripening	fruit ripening, fruit softening and fruit quality	grapes	miR479 *	*BGA*	fruit softing	deep sequencing, bioinformatics analysis	[101]
			miR399 *	*ACO3*			
			miR397 *	*LOX*			
			miR3627 *	*Grip22/PAL*			
			miR2950 *	*CHS*			
			miR22 *	*PE*			
biotic and abiotic stress in fruit	cold response	arabidopsis	*CIL1* (*lncRNA*)	*ROS*	enhances cold stress tolerance	T-DNA insertion mutants	[124]
		orange	miR396b	*GRF*	cold tolerance	ptr-miR396b OE (transgenic lemon (*Citrus limon*))	[119]
		banana	miR393 *	*TIR1/AFB*	cold stress-specific response	bioinformatics analysis	[121]
		mango	*CRlnc26299* * (lncRNA)	*RC12B*	chilling tolerance	Computational Identification	[120]
	salt tolerance	arabidopsis	miR396	*GRF*	salt tolerance	target mimicry (eTM) transgene specific to miR396	[111]
			miR393a/b	*TIR1*	salt stress resistance and ABA signaling pathways	mir393ab double mutant	[109,110]
		pitaya	miR396 *	*GRF*	stress response	bioinformatics analysis	[112]
	heat tolerance	tomato	miR396 *	*GRF*	drought and heat stress	bioinformatics analysis	[125]
		arabidopsis	miR160	*ARF10, ARF16, and ARF17*	heat stress tolerance	eTM-miR160	[116]
		banana	miR156 *	*SPL*	heat stress response	bioinformatics analysis	[121]
		mango	MmiR78769 and MmiR101928 (lncRNA)	phospholipase A and phospholipase D	biotic and abiotic stresses	Computational Identification	[120]
biotic and abiotic stress in fruit	heat tolerance	pear	Novel_188	*Pbr027651.1*	mediate fruit senescence	transient (Novel_188 OE)	[118]
			LNC_000862 * (lncRNA)	miR390a-*Pbr031098.1*	heat tolerance	bioinformatics analysis	[122]
	drought response	arabidopsis	miR396a/b	*GRF*	drought tolerance	35S::*MIR396a* and 35S::*MIR396b*	[127]
			miR159	*MYB101* and *MYB33*	drought tolerance	miR159 OE	[128]
			*DRIR* (lncRNA)	*genes involved in ABA signaling*	Enhances Drought and Salt Stress Tolerance	*DRIR* OE	[129]
		tomato	miR169	*NFYA*	drought and heat stress	STTM-miR169	[126]
			miR159 *	*MYB*		bioinformatics analysis	[125]
			miR160 *	*ARF*			
			miR167 *	*ARF*			
			miR393 *	auxin receptor homologous genes			
	pathogen defense	arabidopsis	miR396	*GRF*	pathogen defense	miR396 target mimics lines	[138]
		apple	Md-miRLn11	*Md-NBS*	pathogen defense	bioinformatics analysis	[132]
		tomato	SlymiR482e-3p	*NBS-LRR*	enhanced resistance to tomato wilt disease	*slymiR482e-3p* KO lines	[133]
			miR156 *	*SPL*	response to ToLCV infections	bioinformatics analysis	[29,135]
			miR159/319	*AP2-like*	viral response (*tomato leaf curl new delhi virus* (tolcndv))	MicroRNA profiling	[136]
			miR172	*TCP, bHLH*			[136]
			LncRNA4504 (lncRNA)	JA signal pathway genes	pathogen defense (*Botrytis cinerea*)	TRV-lncRNA4504	[139]
		pear	pbr-miR156 *	*pbRPS6*	viral defense	bioinformatics analysis	[137]
			pbr-miR164 *	*pbNAC*			
			pbr-miR399 *	*pbTLR*			
			pbr-miR482 *	*pbRX-CC*			

The asterisk (*) represents bioinformatics analysis data.

## 5. Conclusions and Perspective

**Figure 2:**
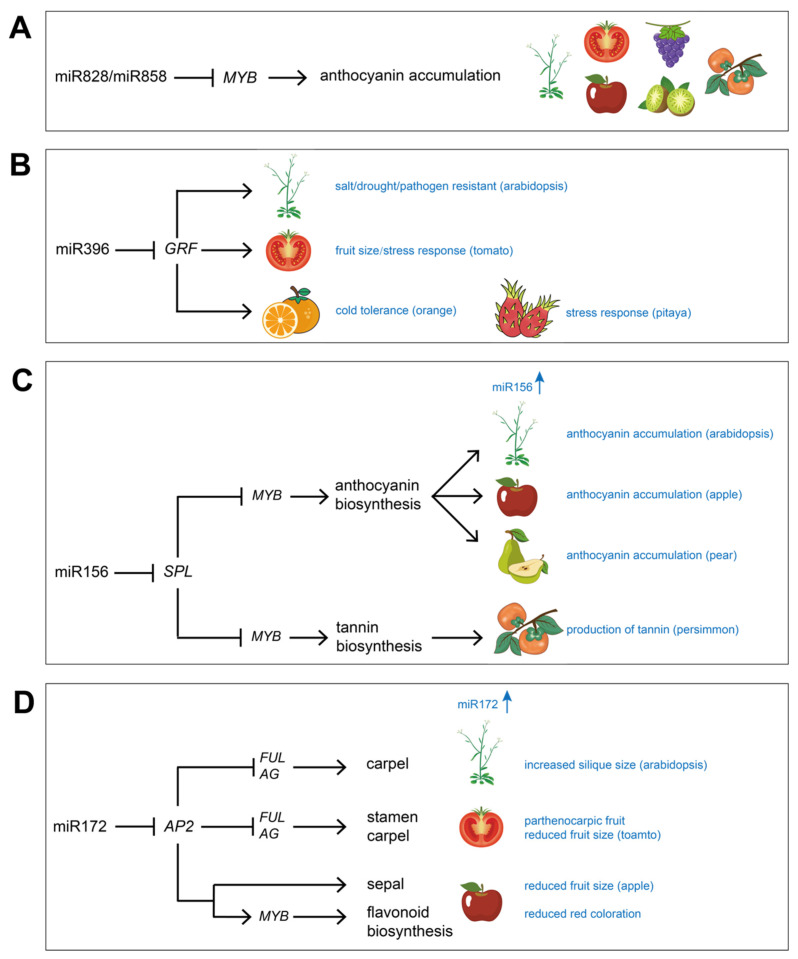
Models of miRNA regulatory modules in fruit crops. (**A**) The miR828/miR858-*MYB* module regulates anthocyanin accumulation in *Arabidopsis*, tomato, apple, grape, kiwifruit and persimmon, respectively. (**B**) The miR396-*GRF* module regulates plant growth and stress response in *Arabidopsis*, tomato, orange and pitaya, respectively. (**C**) The miR156-*SPL* module regulates fruit metabolism in *Arabidopsis*, apple, pear and persimmon, respectively. (**D**) The miR172-*AP2* module regulates fruit size and anthocyanin biosynthesis in *Arabidopsis*, tomato and apple.

It can be inferred that miRNA and lncRNA are important regulators in fruit crops. In both dry and fleshy fruit systems, specific miRNAs and lncRNAs are identified, and their roles are essential in the organization of a functional final organ structure, fruit ripening and stress tolerance (Table 1 and Figure 1). Functional studies have revealed that some miRNA regulatory modules are universally important to fruit crops; for instance, miR828/miR858-*MYB* regulates anthocyanin accumulation, miR396-*GRF* regulates plant growth and stress response, miR156-*SPL* regulates fruit metabolism, and miR172-*AP2* regulates fruit size and anthocyanin biosynthesis (Figure 2). Thus, miRNA-mediated genetic engineering methods could represent an effective approach for the development of superior characters. This is probably an economic way to develop important agricultural traits or innovations by fine-tuning miRNAs instead of suppressing a large number of genes, and it will also allow breeders to obtain a commercially valuable crop species in a short time. 

The involvement of miRNAs in several agriculturally important traits that have been improved to date using CRISPR/Cas9 include crop productivity, quality, and biotic and abiotic stress resistance [140]. For example, generating mutations in *MIR396e* and *MIR396f* increases the grain size and modulates the shoot architecture in rice using a multiplex CRISPR/Cas9-based genome engineering tool [141]. For fruit crops, few studies are available on the CRISPR/Cas9 editing of miRNA family and lncRNA mutants, and much more work is needed to decipher miRNA-mediated regulatory networks. In addition, many genome editing tools are continually developed, thus finding valuable editing switch sites that are important for creating new agronomic traits instead of one or several gene edits. This is a broadly adopted regulatory strategy during plant evolution and it is practical for crop improvement. A growing body of evidence shows the prime editing system applied in plants [142,143,144], suggesting that the prime editing tool would be a promising technology to introduce the desired modification and breed elite crop varieties in fruit crops. We believe that with further understanding of miRNA- and lncRNA-based cellular regulatory networks, additional technologies will emerge for the improvement of fruit agricultural traits.

In particular, a better characterization of the miRNAs and lncRNAs, which can coordinate the main steps of fruit development and ripening in different plant species, may result in the development of novel strategies for fruit crop management. One of the most important challenges in the future will be to functionally analyze more miRNA modules and lncRNA networks in fruit crops, as well as the further achievement of efficient transgene-free genome editing via the CRISPR system in fruit crops. We outline future perspectives in developing miRNA/lncRNA-based breeding strategies for fruit crop improvement and applying genome editing tools for modulating agriculturally important traits.

## Data Availability

The datasets generated during the current study are available at https://www.mdpi.com/ethics (accessed on 24 December 2022).
